# Unsupervised classification of brain-wide axons reveals neuronal projection blueprint

**DOI:** 10.21203/rs.3.rs-3044664/v1

**Published:** 2023-07-03

**Authors:** Diek W. Wheeler, Shaina Banduri, Sruthi Sankararaman, Samhita Vinay, Giorgio A. Ascoli

**Affiliations:** 1 Center for Neural Informatics, Krasnow Institute for Advanced Studies and Bioengineering Department, College of Engineering & Computing, George Mason University, Fairfax VA (USA)

## Abstract

Long-range axonal projections are quintessential determinants of network connectivity, linking cellular organization and circuit architecture. Here we introduce a quantitative strategy to identify, from a given source region, all “projection neuron types” with statistically different patterns of anatomical targeting. We first validate the proposed technique with well-characterized data from layer 6 of the mouse primary motor cortex. The results yield two clusters, consistent with previously discovered cortico-thalamic and intra-telencephalic neuron classes. We next analyze neurons from the presubiculum, a less-explored region. Extending sparse knowledge from earlier retrograde tracing studies, we identify five classes of presubicular projecting neurons, revealing unique patterns of divergence, convergence, and specificity. We thus report several findings: (1) individual classes target multiple subregions along defined functions, such as spatial representation vs. sensory integration and visual vs. auditory input; (2) all hypothalamic regions are exclusively targeted by the same class also invading midbrain, a sharp subset of thalamic nuclei, and agranular retrosplenial cortex; (3) Cornu Ammonis, in contrast, receives input from the same presubicular axons projecting to granular retrosplenial cortex, also the purview of a single class; (4) path distances from the presubiculum to the same targets differ significantly between classes, as do the path distances to distinct targets within most classes, suggesting fine temporal coordination in activating distant areas; (5) the identified classes have highly non-uniform abundances, with substantially more neurons projecting to midbrain and hypothalamus than to medial and lateral entorhinal cortex; (6) lastly, presubicular soma locations are segregated among classes, indicating topographic organization of projections. This study thus demonstrates that classifying neurons based on statistically distinct axonal projection patterns sheds light on the functional organizational of their circuit.

## Introduction.

The classification of neurons in the mammalian nervous system has long been a focus of intensive investigation. While local features from slice preparations in vitro may suffice to infer the circuit roles of GABAergic interneurons^1–3^, long-range projecting axons are crucial architectural elements of neural organization^4,5^ constituting the conceptual and physical nexus between brain-wide circuits and synaptic communication^6^. Thus, projection axons have long been digitally traced from serial sections after in vivo labeling and light microscopy imaging^7–10^. At the same time, their macroscopic extent (~1 cm span; ~1 m cable length) and microscopic caliber (~100 nm branch thickness) combine into a formidable technological challenge for large-scale collection^11,12^. As a result, the number of completely reconstructed projection axons in any mammalian neural system has until recently remained into the low tens.

A source brain region projecting to N targets (where N typically ranges between 10 and 50 in the mouse cortex) could contain any combination of 2^N^-1 distinct axonal projection types. Such a combinatorics challenge requires a large-scale data collection for proper classification. Projects based on fluorescent Micro-Optical Sectioning Tomography (fMOST) technology^13–15^ or the Janelia MouseLight platform^16^, launched in recent years to address this need, produced nearly 10,000 mouse whole-brain single neuron reconstructions registered to a 3D Common Coordinate Framework (CCF)^17^ with consensus anatomical labeling^18^. However, these newly available data do not themselves generate novel scientific insights, explain brain circuitry, or even disprove that axons might simply invade a random subset of the regional target areas^19^. Rigorous methods are needed to test the hypothesis that specific projection types exist, to characterize their identities, and to quantify their population sizes^20^.

This study introduces an original technique to objectively identify projection-based neuronal classes. To ascertain whether a collection of axonal projections might result from essentially random variation within the constraints of regional connectivity or likely reflects distinct neuron types, we begin from the foundational criterion for classification: if a set of items belongs to segregated classes, their pairwise inter-individual differences must be on average larger between than within classes. In other words, two items from the same class should tend to be more similar to each other than two items from separate classes. To implement this logic into a classification framework, we couple rigorous statistical testing with unsupervised hierarchical clustering. A unique strength of this approach is its entirely data-driven granularity: the continuous accumulation of new tracings will progressively refine the classification details with increasing statistical power. We can then characterize the identified projection classes by quantifying their population size, topographic soma distributions, and convergence and divergence patterns.

In the remainder of this article, we first propose a formal definition of and a quantitative solution for the classification problem. We validate our approach by applying it to layer 6 of the primary motor cortex, and then utilize it to study the presubiculum, a rather under-investigated region of the mouse brain. We next quantify the neuronal population sizes of the presubicular projection classes and characterize the spatial distribution of their somata. Finally, we analyze the patterns of divergence and convergence of presubicular projection classes. We conclude by discussing the biological interpretations of these results.

## Results.

### Formal Definition and Quantitative Solution of the Classification Problem.

**Figure 1.**
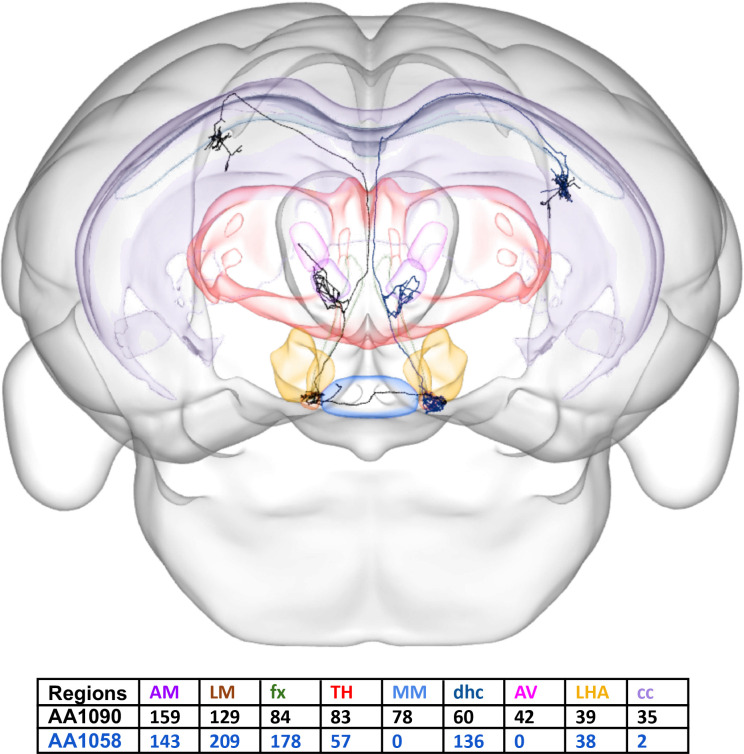
Brain-wide neuronal projections. CCF-registered reconstruction of two presubicular neurons (AA1090 in black and AA1058 in blue from the Janelia MouseLight project) invading 9 regions out of 40 potential targets along with the numbers of axonal points of the neurons in each highlighted region (posterior view of brain). *CCF, common coordinate framework; AM, Anteromedial nucleus; AV, Anteroventral nucleus; cc, corpus callosum; dhc, dorsal hippocampal commissure; fx, fornix; LHA, Lateral hypothalamic area; LM, Lateral mammillary nucleus; MM, Medial mammillary nucleus; TH, other thalamic nuclei.*

**Figure 2.**
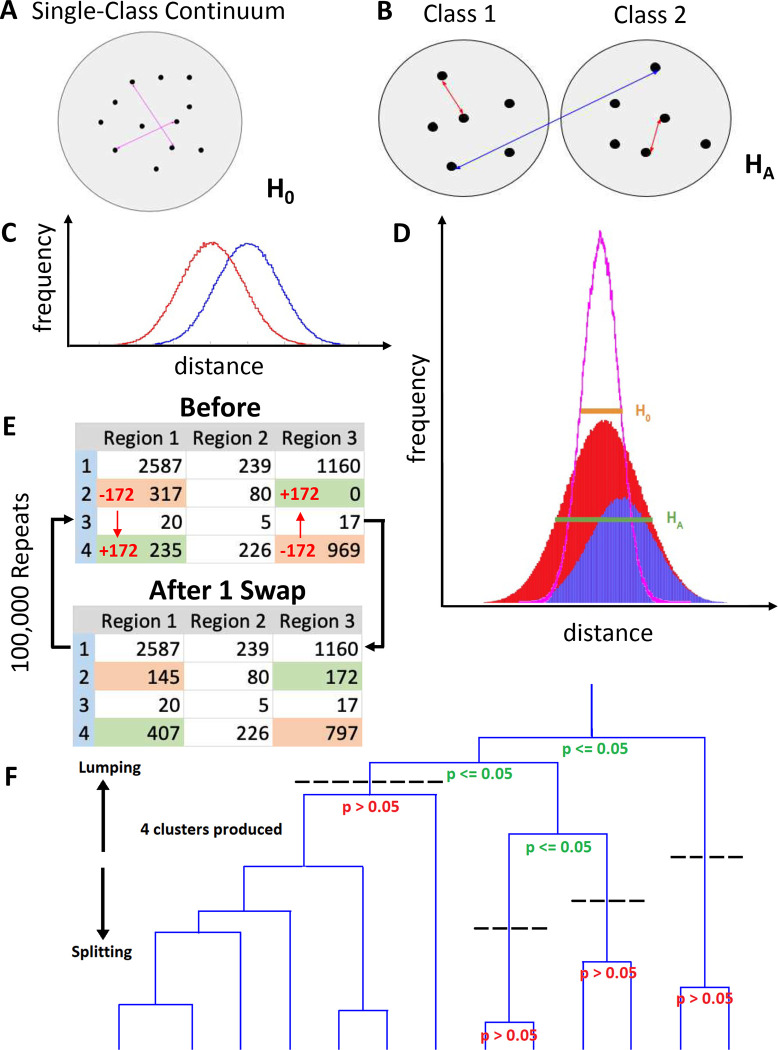
Definitions of neuron classes and clustering methods. (A) In a single-class scenario, the distribution of differences between neurons can be calculated for all neuron pairs (pink double-arrows). (B) If two distinct classes exist, neurons (represented here as black dots) will tend to have more similar projections within their class (red double-arrows) and more different ones across classes (blue double arrow). (C) The differences within the classes (red distribution) will be smaller than those between classes (blue distribution). (D) The distribution of the combined frequency of differences, in a multi-class scenario (red-blue stacked areas; green half-height width), will be wider than that of a single-class distribution (pink curve; orange half-height width). (E) Diagram showing the randomization of projection patterns through the repeated pairwise swapping of axonal point counts between two neurons across two of their potential target regions, which preserves the column (for a given region) and row (for a given neuron) sums of the matrix. This swapping results in a projection pattern “continuum” that matches with the overall distribution that represents the 1-class null hypothesis. (F) Unsupervised hierarchical clustering groups a set of neurons into classes based on their relative pairwise differences or similarities, as modeled by a binary dendrogram. The top (root) of the dendrogram represents all neurons lumped into the same class, while the bottom (leaves) shows all neurons split into separate classes.

The axonal projections of each neuron in a source region can be represented as k-dimensional vectors, where k is the number of target regions invaded by the source region. Each of the k components of the vector quantifies the number of axonal points within the corresponding region (Figure 1). We explore the null hypothesis, H_0_, that all neurons from a source region belong to a single projection class (Figure 2A), as opposed to the alternative hypothesis, H_A_, that distinct projection classes exist from that source region (Figure 2B). If two hypothetical classes exist, the projections will be more similar between neurons within a class and more different across classes (Figure 2C). In such a two-class scenario, the combined within- and across-class distances would thus form a wider distribution than the distribution generated if all neurons belong to just a single class (Figure 2D). To formally test H_A_, we measure all pairwise differences between neurons (as arccosine vector distances, see Methods & Materials). We then generate the distribution of distances for H_0_ by randomizing the projection patterns while preserving total axonal length both by neuron and target region. We achieve this single-class “continuum” by iterative stochastic swapping of axonal points between neurons across two target regions (see Figure 2E and Methods & Materials). We can then apply Levene’s one-tail statistical test to ascertain whether the original distribution of pairwise distances has significantly larger variance than the randomized distribution. If the answer is positive, we must discard H_0_ and accept H_A_. Starting from the top node in an unsupervised hierarchical clustering tree, we can thus repeat Levene’s test on the neurons of each of the two subtrees, continuing the process until none of the variance differences are statistically significant (Figure 2F).

### Validation of the Approach.

**Figure 3.**
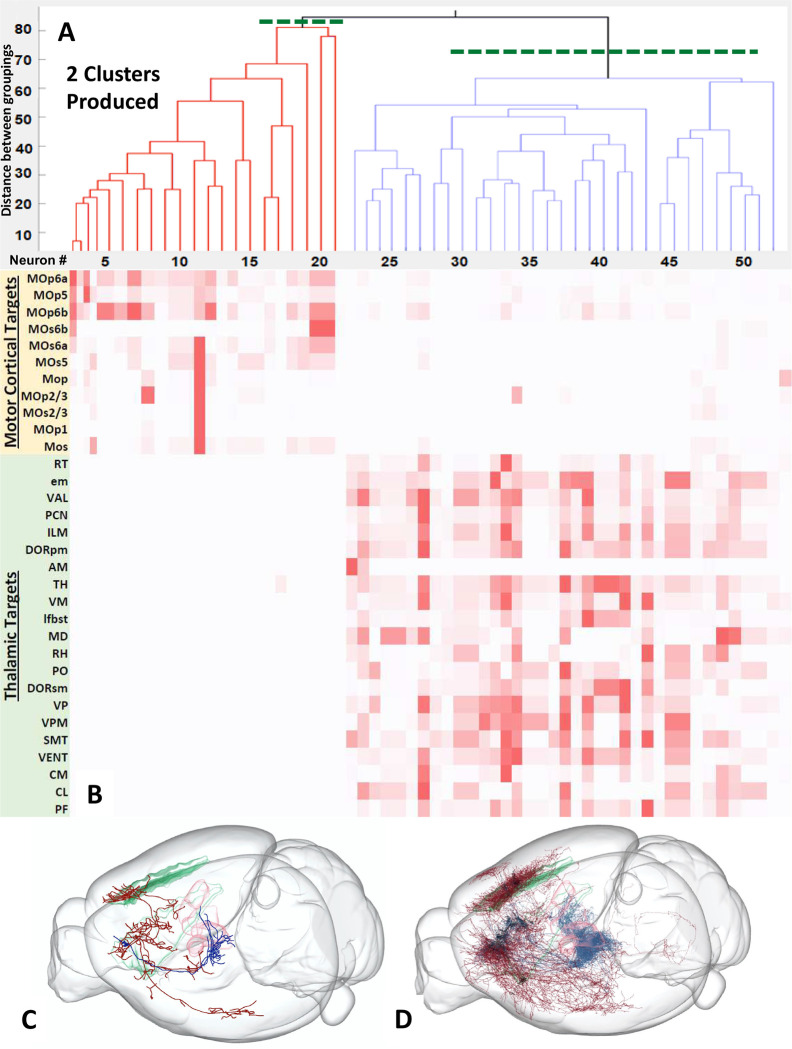
Primary motor cortex L6 (IT vs. CT). (A) Representation of the two clusters produced by Levene’s one-tailed test for the equality of variances and unsupervised hierarchical clustering, using MouseLight neurons from the primary motor cortex, layer 6 (n = 52). (B) Colormap of the axonal distributions of neurons (columns) across anatomical regions (rows), with darker shades representing more axonal projections. (C) Axonal pathways of representative IT (intra-telencephalic, red) and CT (corticothalamic, blue) neurons with semitransparent surfaces of primary motor cortex layer 6 (green) and selected thalamic nuclei (pink). The two black dots indicate the cell body locations of the two representative cells from each class. (D) Axonal pathways of all IT and CT neurons in the MouseLight sample (same color coding).

To validate the above research design, we first analyzed 52 MouseLight layer 6 neurons from the primary motor cortex^21^. This anatomical area is known to contain two distinct projection classes with well-defined subdivisions: cortico-thalamic (CT) and cortico-cortical or intra-telencephalic (IT) neurons^22^. The variance of the distribution of pairwise axonal projection differences of these neurons was significantly larger than that of the randomized projections (p < 0.001; variance of real data = 373.4; variance of randomized data = 195.7), indicating the existence of distinct clusters. However, both subtrees after the first split of unsupervised hierarchical clustering returned a non-significant Levene’s test (IT: p = N/A; variance of real data = 219.9; variance of randomized data = 240.0; CT: p > 0.05; variance of real data = 295.1; variance of randomized data = 264.0), revealing exactly two clusters (Figure 3A). The first cluster, consisting of 21 neurons, projected almost exclusively to motor cortical targets; the second cluster of 31 neurons projected primarily to thalamic targets (Figures 3B–D and Table S1). These patterns were fully consistent with the axonal pathways of the IT and CT neuronal classes, respectively. This finding thus corroborates the validity of employing Levene’s test of variance on pairwise difference distributions to identify statistically distinct classes in unsupervised hierarchical clustering.

### Classification of Projection Neurons from the Mouse Presubiculum.

**Figure 4.**
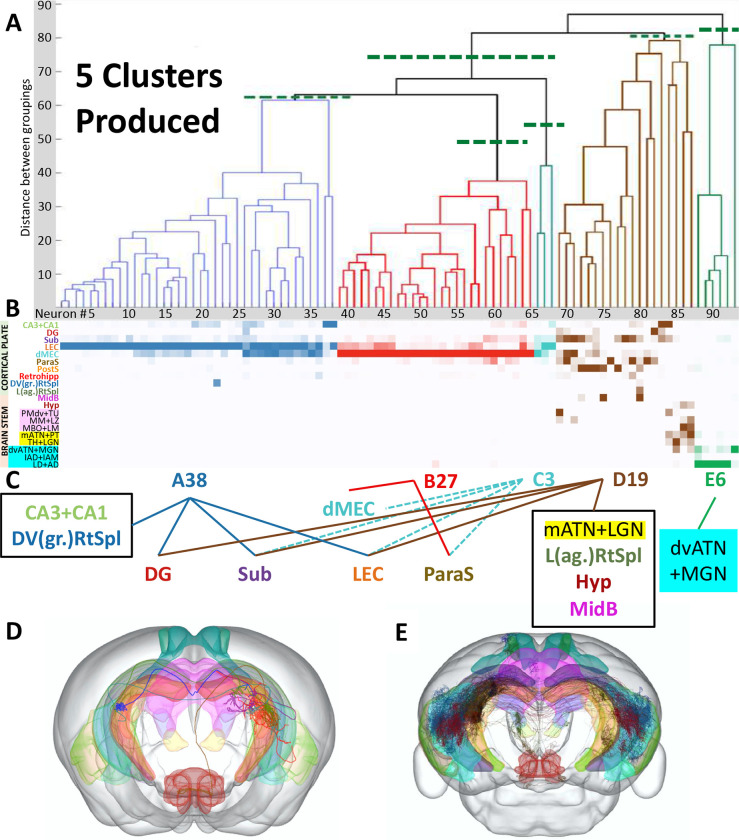
Classification of projection neuron types in the presubiculum. (A) Representation of 5 axonal clusters produced by Levene’s test and unsupervised hierarchical clustering of neurons from the presubiculum (n = 93). (B) Colormap of the axonal distributions of neurons (columns) across anatomical regions (rows), with darker shades representing more axonal projections. Parcel names highlighted in pink are hypothalamus related. Parcel names highlighted in yellow and light blue are thalamus related. (C) Neuron-to-target assignments for the identified axonal projection classes and corresponding anatomical regions (dotted line: contralateral). (D) Anterior view of the mouse brain with one neuron from each class. Color coding of neurons and semitransparent anatomical areas shown in A, B, and C. (E) Posterior view of the brain with all MouseLight presubicular neurons. *CA3*+*CA1: Cornu Ammonis areas 3 and 1; DG: dentate gyrus; Sub: subiculum; LEC: lateral entorhinal cortex; dMEC: dorsal portion of the medial entorhinal cortex; ParaS: parasubiculum; PostS: postsubiculum; Retrohipp: retrohippocampal region; DV(gr.)RtSpl: dorsal and ventral (granular) retrosplenial cortex; L(ag.)RtSpl: lateral (agranular) retrosplenial cortex; MidB: midbrain; Hyp: hypothalamus; PMdv*+*TU: dorsal and ventral premammillary nucleus and tuberal nucleus; MM*+*LZ: medial mammillary nucleus and hypothalamic lateral zone; MBO*+*LM: mammillary body and lateral mammillary nucleus; mATN*+*PT: medial anterior thalamic nucleus and parataenial nucleus; TH*+*LGN: thalamus and lateral geniculate nucleus; dvATN*+*MGN: dorsal and ventral anterior thalamic nucleus and medial geniculate nucleus; IAD*+*IAM: interanterodorsal and interanteromedial nucleus of the thalamus; LD*+*AD: lateral dorsal and anterodorsal nucleus of thalamus.*

We then applied our analytic technique to a lesser-explored source region of the mouse brain: the presubiculum. Unsupervised clustering and the test of variance demonstrated that the 93 MouseLight neurons from the presubiculum form five distinct projection classes (Figures 4A–C). We designate each class by a letter (A-E) followed by the number of neurons in the class (Figure 4C). The first class, A38, primarily targets the lateral entorhinal cortex (LEC), accounting for 82% of axonal extent outside of the presubiculum. This class also invades the dorsoventral (granular) retrosplenial cortex as well as the hippocampal formation (dentate gyrus, CA3, CA2, CA1, and subiculum). The second class, B27, mainly targets the dorsal portion of the medial entorhinal cortex (dMEC), accounting for 92.5% of extra-presubicular axonal extent, as well as retrohippocampal zone and parasubiculum. Class C3 neurons mostly target the contralateral dMEC (42%) and LEC (40%), subiculum (14%), and parasubiculum (4%) through extensive callosal and commissural fibers. Class D19 has the most complex (and unreported) pattern of innervation: in addition to major projections to the subiculum (40.8%) and dentate gyrus (16.3%), it is the sole source of projections to the lateral (agranular) retrosplenial cortex, to the hypothalamus (including the lateral mammillary nucleus and 18 additional nuclei: Table S2), and to the superior and inferior colliculi in the midbrain. This neuronal class also projects to a subset of 8 thalamic nuclei, including the medial part of the anterior thalamic nucleus (ATN) and the lateral geniculate nucleus. Lastly, class E6 projects to a complementary set of 14 other thalamic nuclei, including the ventral, dorsal, anterior, and lateral parts of the ATN and the medial geniculate nucleus. Neurons from all five projection classes also have substantial collaterals within the presubiculum. Examples of projection neurons from each of the presubicular projection classes are depicted in Figures 4D–E.

### Presubicular Projection Classes Have Non-Uniform Neuronal Population Sizes.

Next, we quantified the proportion of neurons in the mouse presubiculum that belong to each projection class. To this aim, we extracted the anterograde tract tracing density distributions from the Allen Institute regional connectivity atlas and matched the fractions of neurons in every class based on their axonal patterns by numerical optimization (see Non-Negative Least Squares in Methods & Materials). The results converged with very small residual error (<0.0006%) indicating a near-exact correspondence between single-neuron and regional projections. Class D19, reaching the midbrain, hypothalamus, lateral (agranular) retrosplenial, and the lateral geniculate (visual thalamus) accounted for a plurality (38.1%) of neurons. Class A38, targeting the hippocampus, subiculum, dorsoventral (granular) retrosplenial cortex, and lateral entorhinal cortex (“what” pathway), accounted for the second largest share (30.6%) of neurons. Class B27, projecting to the parasubiculum and medial entorhinal cortex (“where” pathway) consisted of 16.3% of projection neurons. Class E6, focused on other thalamic nuclei including medial geniculate (auditory), was responsible for 13.7% of presubicular neurons. The diffuse contralateral projections of class C3 comprised the remaining 1.3%.

When accounting for these relative proportions together with the MouseLight axonal projections (Table S2), we can estimate the contribution of each class to the presubicular projections in each collection of target regions. In particular, the dentate gyrus receives 21% of its presubicular afferents from class A38 and 79% from class D19. The subiculum receives 69% of its presubicular afferents from class D19, 30% from class A38, and 1% from class C3. The lateral entorhinal cortex receives 99% of presubicular afferents from class A38 and 1% from class C3. The dorsal medial entorhinal cortex and parasubiculum receive 99% of presubicular afferents from class B27 and 1% from class C3. All other regions are targeted by individual classes: CA3, CA1, and the dorsoventral (granular) retrosplenial cortex by A38; the midbrain, hypothalamus, lateral (agranular) retrosplenial cortex, and part of the thalamic nuclei including mATN and LGN by D19; and the rest of the thalamic nuclei including dvATN and MGN by E6.

### Spatial Distribution of Somata Reveals Topographic Organization of Neuronal Projections.

**Figure 5.**
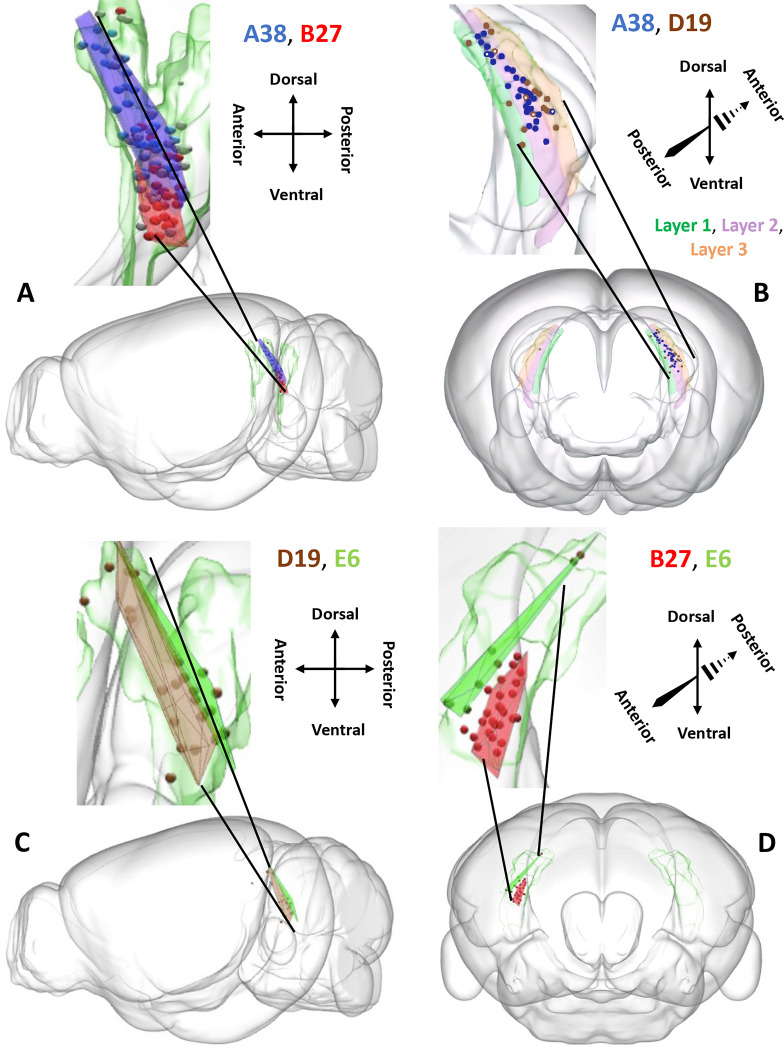
Spatial distributions of somata in the presubiculum across projection classes. Convex hulls of neurons (spheres) from classes A38 (blue), B27 (red), D19 (brown), and E6 (green), and semitransparent presubiculum (green). (A) Left sagittal view of A38 and B27. (B) Layer 1 (green), layer 2 (purple), and layer 3 (orange) of the presubiculum are highlighted in an anterior coronal view, with somata from A38 in blue and D19 in brown. Most of the A38 somata are concentrated in layer 2, while the D19 somata tend to be more concentrated in layers 1 and 3. Somata that do not follow this pattern are indicated with a white dot inside of the circle. (C) Left sagittal view of D19 and E6. (D) Posterior coronal view of B27 and E6.

Computational geometry analysis of soma locations within the presubiculum demonstrated a clear spatial separation among the four main projection classes: A38, B27, D19, and E6 (the smallest class, C3, is largely contralateral projecting). Specifically, the convex hull volume of each neuron class overlapped only minimally (~5–20%) with that of other neuron classes (Figure 5A–C). In particular, class A38 was positioned more rostrally and dorsally relative to the caudal-ventral position of class B27, with approximately 14% of overlap (Figure 5A). The overlap of A38 was maximal with D19 (21%); however, while most A38 neurons had a selective somatic concentration in layer 2 (34/38: 89.5%), D19 had a somatic distribution across all 3 presubicular layers: 21% in layer 1 and 26% in layer 3 (Figure 5B). Class E6 had the most lateral positioning resulting in almost complete segregation from the other projection classes: there were so few overlapping somata that a proper convex hull volume of the overlap could not be calculated (Figures 5C–D).

### Efferent Path Distances from the Same Neurons Significantly Vary by Target Region.

**Figure 6.**
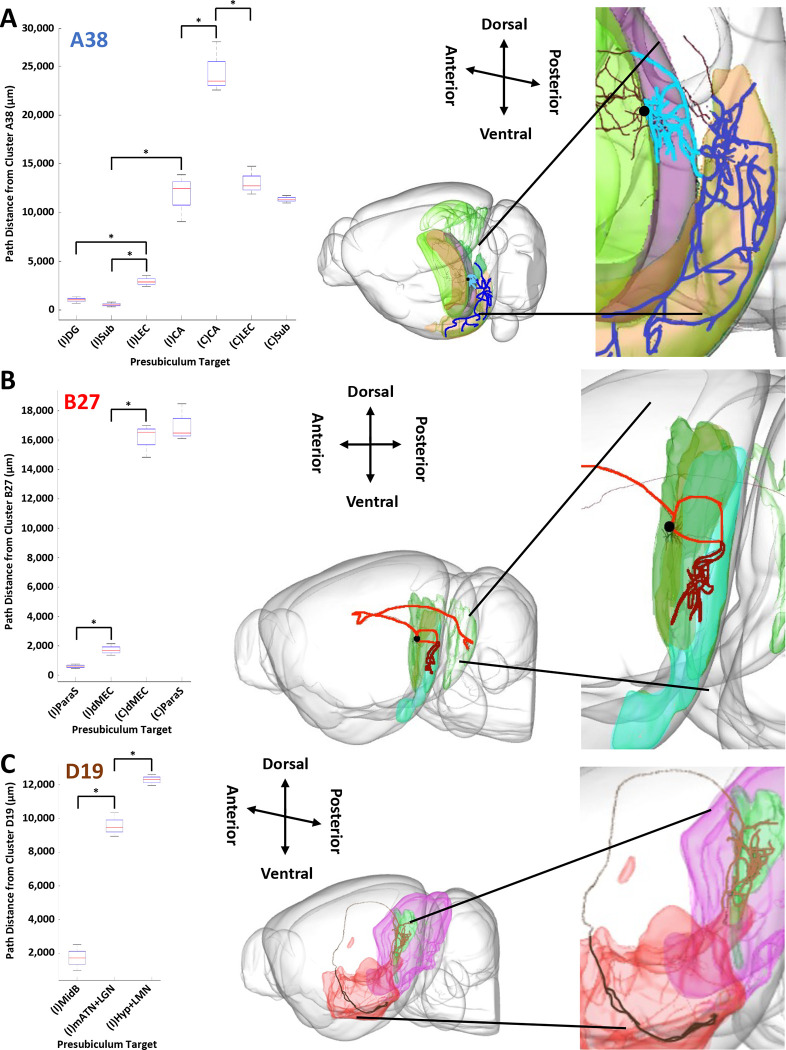
Divergent path distance comparison from one neuron class in the presubiculum to its targets. (A) Left: box and whisker plot depicting the median, first and third quartiles, and full range of the path distances from class A38 to its major ipsilateral (I) and contralateral (C) targets. Right: the path distance of an archetype neuron from class A38 (light blue), from its soma (black) in the ipsilateral presubiculum (green) to the subiculum (purple), is significantly shorter than that (dark blue) to the lateral entorhinal cortex (orange). (B) Left: box and whisker plot depicting the distributions of path distances from class B27 to its major ipsilateral and contralateral targets. Right: the path distance of an archetype neuron from class B27 (light red), from its soma (black) in the ipsilateral presubiculum (green) to the parasubiculum (brown), is significantly shorter than that (dark red) to the medial entorhinal cortex, dorsal zone (cyan). (C) Left: box and whisker plot depicting the path distances from class D19 to its major ipsilateral targets. Right: the path distance of an archetype neuron from class D19 (light brown), from its soma (black) in the ipsilateral presubiculum (green) to the midbrain (magenta), is significantly shorter than that (dark brown) to the hypothalamus and lateral mammillary nucleus (red). See Figure 4 for abbreviation definitions. Significant differences in distances were calculated using a Wilcoxon Signed Rank Test performed on neuronal path distances and multiple testing was corrected for by False Discovery Rate to determine the significance of the resultant p-values.

We tested whether the path distances from presubicular neurons of a given projection class differed across their divergent target regions (Figure 6). In these analyses of divergence, ipsilateral and contralateral targets were considered separately, as the latter are systematically farther than the former. For class A38 neurons, projection distances to the ipsilateral lateral entorhinal cortex, subiculum, and dentate gyrus are shorter than those to the ipsilateral hippocampus; moreover, projection distances to the ipsilateral lateral entorhinal cortex are longer than those to the ipsilateral subiculum and dentate gyrus. Similarly, projection distances to the contralateral subiculum and lateral entorhinal cortex are shorter than those to the contralateral hippocampus. Thus, presubicular efferent path distances differ less between ipsilateral and contralateral hippocampus than between other targets across brain hemispheres (Figure 6A). For class B27, projections to the ipsilateral parasubiculum have shorter paths than those to medial entorhinal cortex, dorsal zone, but the distances are comparable in the contralateral case (Figure 6B). Finally, for class D19, projections both to the ipsilateral medial anterior thalamic nucleus and lateral geniculate nucleus, and to the ipsilateral hypothalamus and lateral mammillary nucleus combined have longer paths than those to the ipsilateral midbrain (Figure 6C).

### Afferent Path Distances to the Same Target Region Significantly Vary by Projection Class.

**Figure 7.**
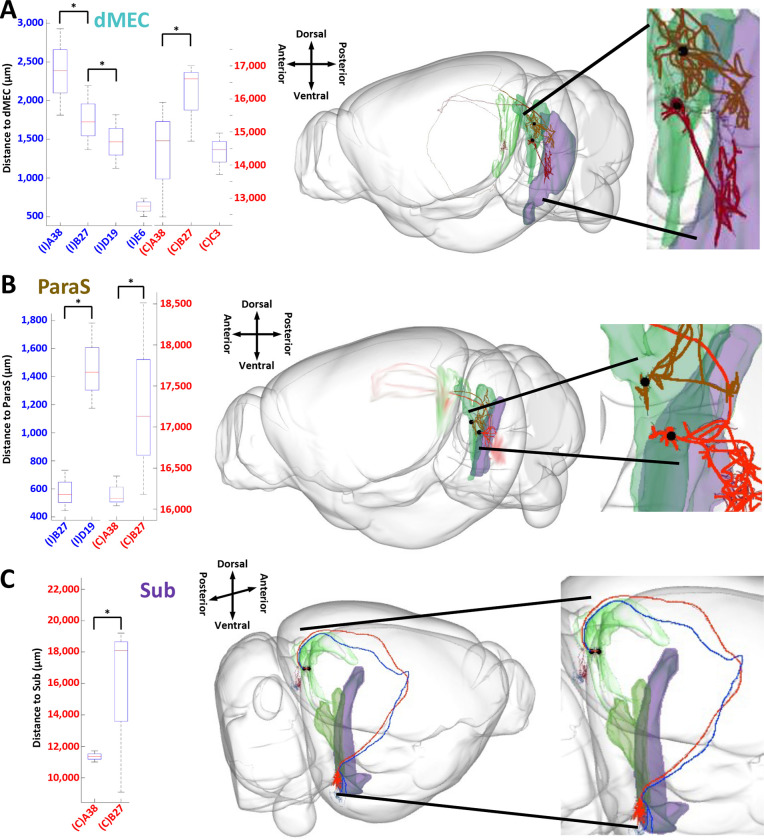
Convergent path distance comparison from each presubiculum cluster to major targets. (A) Top left: box and whisker plot depicting the median, first and third quartiles, and full range of the path distances from neurons in ipsilateral (I) and contralateral (C) classes to the medial entorhinal cortex, dorsal zone. Bottom: the distance of an archetype neuron from class B27 (red), from its soma in the ipsilateral presubiculum (green) to the dMEC (purple), is significantly longer than the comparable distance of an archetype neuron from class D19 (brown). (B) Top left: box and whisker plot depicting the path distances from neurons in ipsilateral and contralateral classes to the parasubiculum. Bottom: the distance of an archetype neuron from class B27 (red), from its soma in the presubiculum (green) to the ipsilateral ParaS (purple), is significantly shorter than the comparable distance of an archetype neuron from class D19 (brown). (C) Top left: box and whisker plot of the path distances from neurons in contralateral classes to the subiculum. Bottom: the distance of an archetype neuron from class A38 (blue), from its soma in the presubiculum (green) to the contralateral Sub (purple), is significantly shorter than the comparable distance of an archetype neuron from class B27 (red). See Figure 4 for abbreviation definitions. Significant differences in distances were calculated using a Wilcoxon Signed Rank Test performed on neuronal path distances and multiple testing was corrected for by False Discovery Rate to determine the significance of the resultant p-values.

Next, we asked whether the axons from neurons of distinct projection classes converging onto their shared targets had different path distances. With the sole exception of the dentate gyrus, all target regions displayed a significant dependence of path distance on the presubicular neuron class (Figure 7A–C). For the ipsilateral medial entorhinal cortex, dorsal zone, projections from E6 and D19 have shorter distances than those from B27 and A38, and projections from B27 have shorter distances than those from A38. For the contralateral medial entorhinal cortex, in contrast, projections from B27 have longer distances than those from A38 (Figure 7A). For the ipsilateral parasubiculum, path distances from D19 are longer than those from B27 (Figure 7B). Finally, for both the contralateral subiculum and parasubiculum, path distances from B27 are longer than those from A38 (Figure 7B–C).

## Discussion.

This study introduced an original method to objectively identify projection-based neuronal classes by pairing the Levene’s test with unsupervised hierarchical clustering. We first conducted a confirmatory study on layer 6 of the primary motor cortex to verify that the proposed technique could reproduce known projection types in a previously explored area of the mammalian brain. The results yielded two clusters with axonal projections consistent with those of the corticothalamic and intratelencephalic neuron classes found in past studies, thereby confirming the validity of the technique^23^.

To test whether the technique could lead to novel insights, we then applied it to the presubiculum, a region with crucial cognitive function^24^, yet few studies on its circuitry^25^. The results yielded five clusters, indicating distinct neuron classes, which led us to reject the null hypothesis that projection neurons exhibit random variation within the constraints of regional connectivity from the presubiculum. In an earlier study^26^, retrograde tracing identified five classes of neurons projecting from the presubiculum, which target the retrosplenial cortex, contralateral subiculum, medial entorhinal cortex, anterior thalamic nucleus, and lateral mammillary nucleus. Our results confirm the existence of these five classes and add new information that reveals patterns of divergence (e.g., class A38 projects to the retrosplenial cortex, dentate gyrus, subiculum, and entorhinal cortex), convergence (e.g., the subiculum receives projections from classes A38, contralateral C3, and D19), and specificity (e.g., class E6 projects exclusively to the medial geniculate nucleus, and all hypothalamic regions receive projections solely from class D19).

The proposed clustering technique correctly distinguishes cortical (classes A38, B27, and C3) from subcortical (D19 and E6) pathways in the second binary split in the hierarchical classification. These results also add cellular level details to previously reported presubicular projections to retrosplenial cortex and thalamic reticular nuclei^27^, as well as a broader circuit context to the characterization of individual presubicular neurons targeting the medial entorhinal cortex^28^.

Furthermore, our findings reveal that several target regions are spatially subdivided according to the differing inputs between classes. These regions include the entorhinal cortex (lateral projections mainly from class A38 and medial projections primarily from class B27), retrosplenial cortex (dorsoventral granular projections almost exclusively from class A38 and lateral agranular projections solely from class D19), and thalamus (medial anterior thalamic nucleus and lateral geniculate nucleus projections principally from class D19 and dorsoventral anterior thalamic nucleus and medial geniculate nucleus projections predominantly from class E6). Some of these regional subdivisions also have known functional distinctions: for instance, the medial entorhinal cortex specializes in spatial representation while the lateral entorhinal cortex specializes in integrating sensory input^29^. Among the thalamic geniculate nuclei, the medial geniculate nucleus is part of the auditory pathway, whereas the lateral geniculate nucleus is part of the visual pathway^4^.

From a comparison of divergent path distances from one presubicular class to its major targets, along with a comparison of convergent path distances from each presubicular class to collectively major targets, we found that path distances to the same targets were significantly different between classes, as were the path distances to distinct targets within most classes. This might imply that electrical impulses reach different targets with varying delays, both within the same class and between classes.

Topographic analysis of presubicular classes revealed spatial separation between the somata of each class. This suggests the possibility of anatomically mapping the input and output of the circuitry specializing in head direction computations^30^. Our reported topography of presubicular projections classes is consistent with the recently observed local modularity of the head-direction microcircuit^31^, and may help clarify the relationship between the egocentric and allocentric spatial and episodic representations of the cortico-hippocampal system^32^.

As with many secondary data analyses, we have limited knowledge of, and control over, artifactual shortcomings in the utilized datasets due to possible idiosyncrasies in labeling, imaging, tracing, registration, and mapping. However, the technique introduced with this work is applicable to many disparate sources of data besides MouseLight, including fMOST^13–15^ and even MapSeq/BarSeq^33,34^. These data sources follow separate experimental and computational protocols, allowing independent validation for the source regions in which these datasets overlap. Our results so far, in the cases of the mouse primary motor cortex and presubiculum, indicate that the executed analysis is robust to these possible confounding variables^22^.

## Conclusion.

Overall, this study revealed that neurons can be divided into distinct classes based on axonal projection patterns, as demonstrated in layer 6 of the primary motor cortex and the presubiculum. Our applied analyses can be used to similarly analyze neurons projecting from all other mouse brain regions with sufficient data. There are currently approximately 40 regions fitting this criterion in the existing datasets, but this number is expected to grow in the near future. Furthermore, we suggest the application of pairing Levene’s test and unsupervised hierarchical clustering to other complementary datasets, such as single-cell transcriptomic datasets, to classify neurons across a molecular domain, in addition to an anatomical domain, as demonstrated here. Moreover, all these complementary datasets are broadly expected to continue to grow in sample size, brain coverage, and acquisition pace^35,36^, supporting a call to establish cloud-based, community accessible pipelines for robust, rigorous, and systematic neuronal characterization^37,38^.

## Material & Methods.

### Data Extraction, Storage, and Conversion.

The location of each axonal data point for nearly 1100 neurons was extracted from the Janelia MouseLight public dataset^21^ using the freeware JSONLab v1.5 (https://www.mathworks.com/matlabcentral/mlc-downloads/downloads/submissions/33381/versions/22/download/zip). These data were contained in a JSON file for each neuron, where X-Y-Z coordinates and parcel information were provided for each axonal point of the neuron. The axonal points in each brain parcel were tabulated for all neurons and were stored in a matrix (Tables S1–2), in which each row represents a neuron, each column represents a parcel, and the values in each cell represent the axonal counts of a particular neuron in a particular region (Figure 1).

### Hypothesis Design.

To determine whether distinct projection classes of neurons exist from a particular parcel of the brain, hypothesis H_A_, we tested the pairwise differences between neurons from the experimental matrices described above. If only a single class of neurons exists, then only a single distribution of differences between neurons will be generated (Figure 2A). If two hypothetical classes exist, then the differences between neurons, evaluated two at a time, will be smaller within a given class than across the two classes (Figure 2B–C). In a multi-class scenario, a histogram of the differences between neurons should be wider than the distribution generated when all the neurons belong to just a single class (Figure 2D). To generate the distribution of differences for the null hypothesis, H_0_, a randomized control matrix was generated from the original experimental matrix through multiple iterations of the stochastic pairwise swapping of axonal counts from two neurons across two target regions (Figure 2E). This method randomized the projection patterns, yielding a “continuum” consistent with the regional connectivity of Figure 2A, while preserving axonal sizes (row sums) and regional targeting (column sums) of the original experimental matrix.

### Levene’s Test.

We assessed the hypothesis that the variance of experimental data was significantly larger than the variance of randomized data (α = 0.05). For both the experimental and randomized matrices, we computed the arccosine between a pair of neuronal vectors, each composed of the axonal counts across all target regions (https://github.com/Projectomics/MATLAB). These angles measure the projection difference of two neurons across all brain parcels. We then performed a 1-tailed Levene’s test^39^ on the angle distributions of the experimental and randomized matrices to assess whether their variances differed significantly. To this aim, we used the MATLAB function vartestn with the TestType parameter set to LeveneAbsolute. If the experimental data had a greater variance than the randomized data, then the experimental data could be further divided into classes, consistent with the scenario presented in Figure 2B.

### Unsupervised Hierarchical Clustering.

We used unsupervised hierarchical clustering to determine a biologically accurate division of neuron classes based on axonal projection patterns. Specifically, the MATLAB linkage function, with the “average” algorithm for computing distance between clusters, was utilized on the 93 MouseLight neurons originating in the presubiculum and the 52 MouseLight neurons originating in layer 6 of the primary motor cortex. The initial assumption (null hypothesis) was that all neurons were part of a single class. If Levene’s test yielded significant results, the number of class divisions was incremented, and the technique was again repeated on each class division. This iterative process continued until none of the subdivided classes yielded significant results, thereby yielding the final class divisions (Figure 2F).

### Non-Negative Least Squares.

To estimate the fractional counts of cells in each of k projection classes in each region, we matched their respective single-cell axonal patterns against the regional connectivity from anterograde tracing to the m known targets, as presented in the Allen Mouse Brain Connectivity Atlas (http://connectivity.brain-map.org/projection). The problem is equivalent to a set of constrained, weighted, linear equations that can be solved numerically by non-negative least-square (NNLS) optimization^40^. NNLS finds the values x that minimizes the Euclidean norm of (Ax − b) with the constraint x ≥ 0^41^, where x is the k-dimensional vector representing the fractions of neurons in each class; b is the m-dimensional vector representing the weights of the regional projections to each target; and A is a k-by-m matrix with rows representing the projections of each class (the sum of the summary vectors of the corresponding neurons) and columns representing target regions. NNLS was computed using the lsqnonneg function in MATLAB.

Matrix A and vector b were based on data from the MouseLight dataset (Table S2) and the Allen Mouse Brain Connectivity Atlas, respectively. Setting the target region to the whole brain in the Connectivity Atlas and the source region to the presubiculum resulted in 7 tracing experiments, which included projection volumes and projection densities for each target brain region. Cross referencing the targeted regions of the MouseLight axonal projections with target regions that appeared in all 7 anterograde tracing experiments resulted in a listing of 66 regions. Matrix A was created with rows representing these 66 brain regions and columns representing the 5 neuron classes found by pairing Levene’s test with unsupervised hierarchical clustering of the presubiculum data (Table S3). The average projection volume and density values for each of the 66 regions were calculated from the 7 experiments, and the averages were multiplied to populate the columns of vector b.

To obtain the highest confidence in the NNLS analysis, matrix A was sequentially “bi-normalized” first by axonal length and then by invaded region. Specifically, first each cell in matrix A was normalized so that each row summed to one. Next, each value was divided by the number of regions, 66, and multiplied by the number of clusters, 5, such that the sum of all values in matrix A equaled 5. Subsequently, each cell in matrix A was normalized so that each column summed to one. Vector b was normalized such that the sum of all values equaled to one. Finally, the squared Euclidean norm of the residual of the MATLAB function lsqnonneg was calculated as a proxy for the uncertainty of the analysis.

### Soma Analysis.

To quantify the spatial separation among the somata among the neuron projection classes in the presubiculum, we performed a convex hull analysis for the location of the soma centers in each class using MATLAB. To create the convex hull, outliers were removed by iteratively going through all points in each class and calculating the volume of the convex hull without each point. If the volume differed by more than 1/n of the volume of the original convex hull, which included all points, the point was considered an outlier and removed from the dataset. This established an algorithmic thresholding that corresponded well with the visual inspection of potential outliers. However, if removing the outliers resulted in fewer than four somata, the minimal number of points required to conduct a convex hull analysis, all points were considered. Between each pair of convex hulls, the proportion of the volume of overlap to the volume of the union of the convex hulls was used to assess the similarity between topographic locations.

### Analysis of Divergence and Convergence.

Utilizing the original JSON data files, for every neuron in each presubiculum class, we measured the path distance from the soma to each axonal point in the target region. We then calculated the median path distance to each target region across all neurons in the class, and performed a Wilcoxon Signed Rank Test^42^, using the MATLAB function ranksum, to assess whether the path distances to each characteristic target of a particular class were significantly different. Using the same data files, we also performed a Wilcoxon Signed Rank Test to assess whether the path distances to each characteristic target between all clusters were significantly different. In both sets of comparisons, multiple testing was corrected for by False Discovery Rate to determine the significance of the resultant p-values.
